# Impacts of solid fuel use versus smoking on life expectancy at age 30 years in the rural and urban Chinese population: a prospective cohort study

**DOI:** 10.1016/j.lanwpc.2023.100705

**Published:** 2023-02-13

**Authors:** Qiufen Sun, Dong Sun, Canqing Yu, Yu Guo, Dianjianyi Sun, Pei Pei, Ling Yang, Yiping Chen, Huaidong Du, Dan Schmidt, Rebecca Stevens, Kai Kang, Junshi Chen, Zhengming Chen, Liming Li, Jun Lv, China Kadoorie Biobank Collaborative Group

**Affiliations:** a Department of Epidemiology & Biostatistics, School of Public Health, Peking University, Beijing, 100191, China; b Peking University Center for Public Health and Epidemic Preparedness & Response, Beijing, 100191, China; c Fuwai Hospital Chinese Academy of Medical Sciences, Beijing, China; d Medical Research Council Population Health Research Unit at the University of Oxford, Oxford, United Kingdom; e Clinical Trial Service Unit & Epidemiological Studies Unit (CTSU), Nuffield Department of Population Health, University of Oxford, United Kingdom; f NCDs Prevention and Control Department, Henan CDC, Zhengzhou, Henan, China; g China National Center for Food Safety Risk Assessment, Beijing, China

**Keywords:** Solid fuel use, Household air pollution, Life expectancy, Chinese population, Health equity

## Abstract

**Background:**

The impact of solid fuel use on life expectancy (LE) in less-developed countries remains unclear. We aimed to evaluate the potential impact of household solid fuel use on LE in the rural and urban Chinese population, with the effect of smoking as a reference.

**Methods:**

We used data from China Kadoorie Biobank (CKB) of 484,915 participants aged 30–79 free of coronary heart disease, stroke, or cancer at baseline. Analyses were performed separately for solid fuel use for cooking, solid fuel use for heating, and smoking, with participants exposed to the other two sources excluded. Solid fuels refer to coal and wood, and clean fuels refer to electricity, gas, and central heating. We used a flexible parametric Royston-Parmar model to estimate hazard ratios of all-cause mortality and predict LE at age 30.

**Findings:**

Totally, 185,077, 95,228, and 230,995 participants were included in cooking-, heating-, and smoking-related analyses, respectively. During a median follow-up of approximately 12.1 years, 12,725, 7,531, and 18,878 deaths were recorded in the respective analysis. Compared with clean fuel users who reported cooking with ventilation, participants who used solid fuels with ventilation and without ventilation had a difference in LE (95% confidence interval [CI]) at age 30 of −1.72 (−2.88, −0.57) and −2.62 (−4.16, −1.05) years for men and −1.33 (−1.85, −0.81) and −1.35 (−2.02, −0.67) years for women, respectively. The difference in LE (95% CI) for heating was −2.23 (−3.51, −0.95) years for men and −1.28 (−2.08, −0.48) years for women. In rural men, the LE reduction (95% CI) related to solid fuel use for cooking (−2.55; −4.51, −0.58) or heating (−3.26; −6.09, 0.44) was more than that related to smoking (−1.71; −2.54, −0.89). Conversely, in urban men, the LE reduction (95% CI) related to smoking (−3.06; −3.56, −2.56) was more than that related to solid fuel use for cooking (−1.28; −2.61, 0.05) and heating (−1.90; −3.16, −0.65). Similar results were observed in women but with a smaller magnitude.

**Interpretation:**

In this Chinese population, the harm to LE from household use of solid fuels was greater than that from smoking in rural residents. Conversely, the negative impact of smoking was greater than solid fuel use in urban residents. Our findings highlight the complexity and diversity of the factors affecting LE in less-developed populations.

**Funding:**

10.13039/501100001809National Natural Science Foundation of China, National Key R&D Program of China, 10.13039/501100017647Kadoorie Charitable Foundation, UK 10.13039/100010269Wellcome Trust.

**Research in context**

**Evidence before this study**

We searched PubMed, EMBASE, and Google Scholar for articles published from the inception of each database to September 31, 2022, using a combination of terms: ("life expectancy" OR "life years" OR "life-years lost" OR "years of life lost" OR "all-cause mortality" OR "mortality" OR "death") AND ("solid fuel use" OR "coal" OR "wood" OR "biomass" OR "indoor air pollution" OR "household air pollution"). We implemented no restriction on study type or language. Relevant studies were also found by checking reference lists of identified articles. Although a few epidemiological studies have demonstrated that household use of solid fuels was associated with increased risks of morbidity and mortality from chronic disease, how much of the years of life lost can be attributable to solid fuel use in less-developed countries remains unknown. Our previous study showed that five low-risk lifestyle factors, including non-smoking, were associated with longer life expectancy (LE) for Chinese adults, but the estimates were lower than that of the European and American populations. We hypothesized that many other factors could also affect LE in less-developed countries, such as the environmental hazards in the home, work, and broader outdoor environment.

**Added value of this study**

The findings of this study show that household use of solid fuels for cooking or heating was associated with lower LE in the Chinese population. Using solid fuels for cooking without ventilation and heating with solid fuels had roughly similar impacts on LE as smoking. The harm to LE from household use of solid fuels was greater than that from smoking in rural residents. Conversely, the negative impact of smoking was greater than solid fuel use in urban residents. The present study highlights the complexity and diversity of the factors affecting LE in less-developed countries. Besides the lifestyle risk factors of global health importance, such as smoking, factors unique to less-developed populations, like solid fuel use, should not be ignored.

**Implications of all the available evidence**

Household solid fuel use is one of the major threats to LE in less-developed areas. Boosting the infrastructure of clean energy supply is urged to improve health outcomes and health equity.

## Introduction

Incomplete combustion of solid fuels generates a large amount of pollutants, such as fine particulate matter (PM_2.5_), that can penetrate deep into the lungs and enter the bloodstream. Globally, an estimated 2.4 billion people are using solid fuels for domestic purposes like cooking, mainly in less-developed countries, including China, where an estimated 293 million people still heavily rely on solid fuels.^1^ Using risk estimates of PM_2.5_ with all-cause mortality, the latest report from the Global Burden of Disease Study estimated that 23.1 million premature deaths were attributable to indoor air pollution caused by the combustion of solid fuels in 2019, among which 1.26 million deaths occurred in China.^2^

A few epidemiological studies have demonstrated that household use of solid fuels was associated with increased risks of morbidity and mortality from chronic diseases.^3^, ^4^, ^5^ However, the commonly used relative indicators, like relative risk, are vague conceptions for laypeople. In contrast, being an absolute measure, life expectancy (LE) is more intuitive and has become a common metric for establishing public health priorities. So far, there is scarce quantitative research assessing how much of the years of life lost could be attributable to solid fuel use. Only an ecological study in Sub-Saharan Africa reported that increased PM_2.5_ concentration from household combustion was associated with lower LE, but the result could be subjected to ecological fallacy.^6^ Our previous study showed that five low-risk lifestyle factors, including never smoking, were associated with longer LE for Chinese adults, but the estimates were lower than that of the European and American populations.^7^ We hypothesized that many other factors could also affect LE in less-developed countries, such as the environmental hazards in the home, work, and broader outdoor environment.^1^

The present study aimed to evaluate the potential impact of household solid fuel use on LE at age 30 in the Chinese population, with the effect of smoking as a reference. We further examined whether there was an urban-rural difference in the impact of solid fuel use and smoking on LE. This study, together with our previous findings,^7^ will help to comprehensively understand the factors that may influence the LE of the Chinese population.

## Methods

### Study design and participants

The China Kadoorie Biobank (CKB) study is a nationwide population-based prospective cohort study. Details of the study design have been previously reported.^8^ In brief, during the 2004-08 baseline survey, 512,723 participants aged 30–79 were recruited from five urban and five rural areas. Information collected at baseline was recorded using an electronic questionnaire system incorporating built-in functions to avoid missing items and minimize logic errors. All participants signed an informed consent form. Ethical approval was obtained from the Ethics Review Committee of the Chinese Center for Disease Control and Prevention (CDC; Beijing, China) and the Oxford Tropical Research Ethics Committee, University of Oxford (UK).

**Fig. 1:**
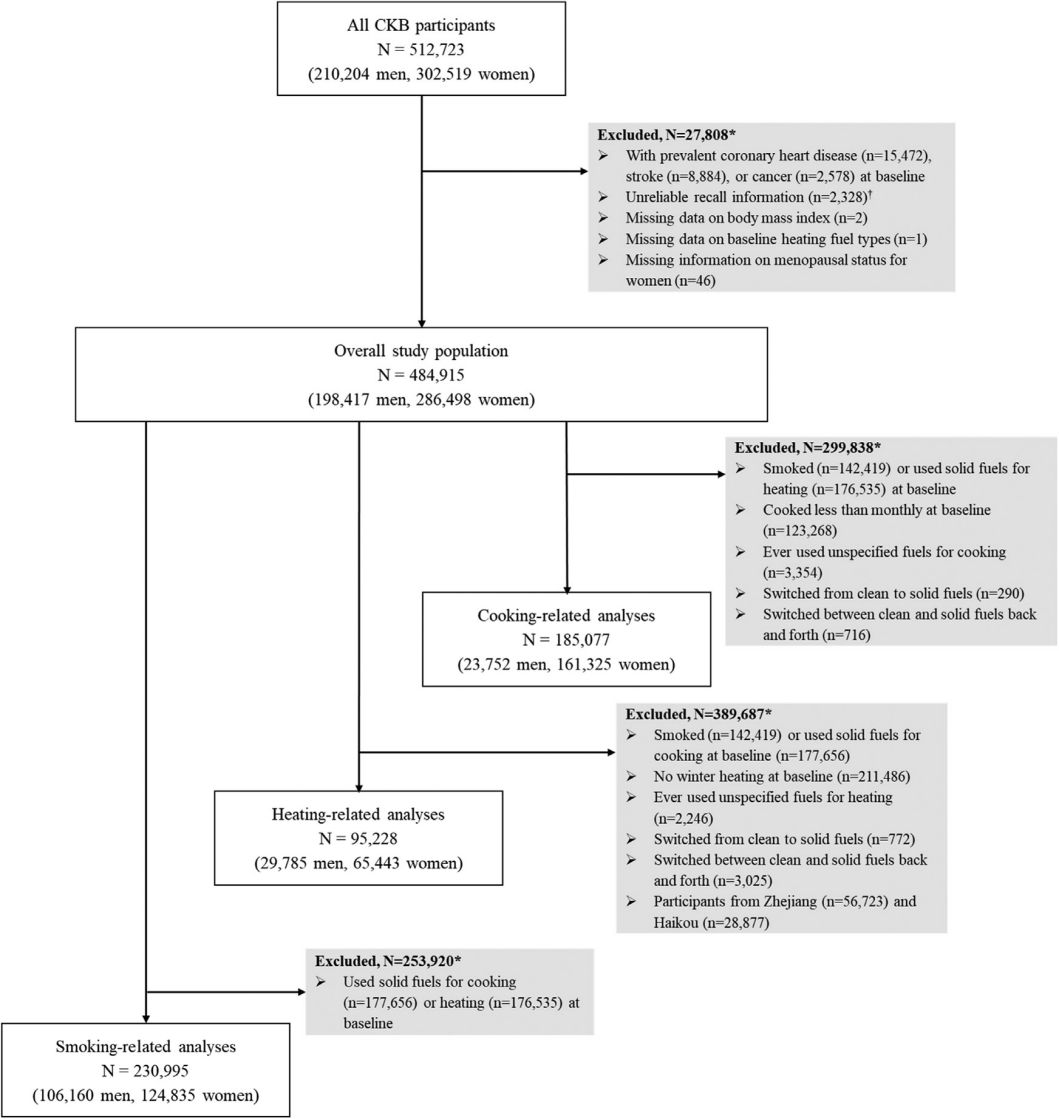
**Flow chart of participants included in the study**. ∗Reasons for exclusion were not mutually exclusive. ^†^The cumulative duration of the last three residences exceeded the baseline age by greater than 1 year.

The flow chart of the exclusion of the participants is shown in Fig. 1. Participants with prevalent coronary heart disease (n = 15,472), stroke (n = 8884), or cancer (n = 2578) at baseline were excluded to minimize the reverse causality bias resulting from smoking cessation, change in cooking behavior, or fuel choice. We also excluded participants with unreliable recall information and missing data on variables of interest, yielding 484,915 participants. We considered three sources of respiratory irritants in this study: solid fuel use for cooking, solid fuel use for heating, and tobacco smoking. We further applied different exclusion criteria to analyze them separately.

When analyzing one of the exposures, we excluded participants exposed to the other two sources to avoid potential confounding. Specifically, participants who used solid fuels for cooking (n = 177,656) or heating (n = 176,535) or smoked (n = 142,419) at baseline were excluded as appropriate. We further excluded the following participants in cooking- and heating-related analyses, respectively: (1) those who cooked less than monthly (n = 123,268) or with no winter heating at baseline (n = 211,486); (2) those who ever used unspecified fuels, switched from clean to solid fuels, or switched between clean and solid fuels back and forth; (3) those from Haikou and Zhejiang in heating-related analyses, because few participants reported winter heating in these two regions (n = 355, <1%).

### Assessment of exposure and covariates

Exposure assessments for fuel use and tobacco smoking have been described in detail elsewhere.^5^^,^^9^ Each participant was asked to recall the duration (in years) of residence, cooking frequency, and winter heating for up to three most recent residences. Participants who reported cooking at least monthly or heating in winter were further asked for the primary fuel type used, which included electricity, gas, coal, wood, or other unspecified fuels, plus central heating (a system generating heat centrally and carrying it to individual households by water or steam through pipes). If multiple fuel types were used simultaneously, only the one used most frequently was recorded. Gas, electricity, and central heating were classified as clean fuels, and wood and coal were classified as solid fuels. For residences with a cooking facility, we further asked if ventilation facilities were present (for all stoves, not all stoves, none).

Based on the primary fuel used in the baseline residence, participants were classified as solid fuel users (coal or wood as separate exposures or combined into a single solid fuel exposure group) or clean fuel users. For cooking-related analysis, we further constructed a four-category composite exposure to explore the impact of ventilated cookstove use, including clean fuel use with ventilation, clean fuel use without ventilation, solid fuel use with ventilation, and solid fuel use without ventilation.

For smoking, ever-smokers were asked to report the frequency, type, and amount of tobacco smoked daily. Former smokers were additionally asked for years since stopping smoking and the reason for quitting. Since half of the former smokers in the CKB cohort quit smoking due to illness,^10^ we included former smokers who stopped smoking for illness in the current smoker category to avoid the misleadingly elevated risk in former smokers.

Baseline covariates information inquired by questionnaire included sociodemographic characteristics (age, region, highest education, marital status, occupation, and household income), lifestyle (alcohol consumption, physical activity, dietary habits), environmental tobacco smoke, and women's reproductive information. The total amount of pure alcohol consumed in grams was estimated based on the self-reported beverage type and volume drunk.^11^ For physical activity, participants were asked about the usual type and duration of activities in occupational, commuting, domestic, and leisure-time-related domains in the past year. The metabolic equivalent of task-hours per day (MET-h/d) was calculated by multiplying the MET value of each type of activity and the hours spent on that activity per day and then summarizing across all activities.^12^ Trained staff measured height and weight using standard instruments and protocols. Body mass index (BMI) was calculated as weight in kilograms divided by height in meters squared.

### Ascertainment of deaths

Participants were followed up for mortality from baseline recruitment until death, loss to follow-up, or 31 December 2018 (for the present analysis), whichever came first. The vital status and date of death were ascertained from the local death registry using a unique national identification number.^13^^,^^14^ The follow-up was supplemented with annual active follow-up for those who failed to link to the local health insurance system, meaning no way to identify their status, to minimize loss to follow-up.

### Statistical analysis

All analyses were performed for cooking, heating, and smoking and for men and women separately. Baseline characteristics of the study population were presented as means (SD) or numbers (percentage) by categories of cooking and heating fuel types and smoking status, with adjustment for age, sex, and study area, as appropriate.^15^

We used a Kaplan–Meier survival curve to compare survival probabilities after baseline enrolment between different exposure categories. Hazard ratios (HRs) and 95% confidence intervals (CIs) of all-cause mortality were estimated using a flexible parametric Royston-Parmar proportion-hazards model, with age as time scale. Royston-Parmar model uses restricted cubic splines to model the baseline cumulative hazard that permits the continuous estimation of absolute measures of effect, such as survival probability.^16^^,^^17^ The proportional hazards assumption was assessed graphically by plotting log–log survival functions for each category, and no violation was observed. Several potential confounders were adjusted, including age at baseline, study areas, education, marital status, occupation, household income, cookstove with ventilation, passive smoking, alcohol consumption, physical activity, dietary factors (intake frequency of fresh fruits, fresh vegetables, and red meat), BMI, and menopausal status (only in women). Cooking and heating fuel exposures and smoking status were mutually adjusted.

The calculation of years of life lost (i.e., the difference in average LE) involved third steps. First, the survival curve was predicted for each individual based on the parametric model and averaged over all individuals. Second, residual LE was estimated as the area under the survival curve by integrating the curve up to age 100, conditional on surviving at ages 30 to 100 (1-year intervals). Third, the years of life lost and 95% CIs were calculated as the difference between the areas under the survival curves of different exposure categories of interest. Details of the calculation method have been described in the appendix (p3-4).

Sensitivity analyses were performed to test the robustness of the results for cooking and heating-related analyses: (1) excluding participants who had stopped using solid fuels for <10 years from the referent group; (2) excluding participants from study areas where solid fuels were rarely used, including Qingdao (0.47%) and Harbin (1.50%) for cook-related analysis and Suzhou (0.05%) for heating-related analysis.

Considering the distinct urban-rural differences in fuel choice and smoking habits, we conducted subgroup analyses by residence. To investigate the potential joint effects of any two exposures, we derived two-by-two composite exposures of cooking fuel use (clean fuels, solid fuels), heating fuel use (clean fuels, solid fuels), and smoking status (never smoker, ever smoker). All statistical analyses were performed using Stata (version 15.0, StataCorp). Graphs were plotted using R version 4.0.3.

### Role of the funding source

The funders had no role in the study design, data collection, data analysis and interpretation, writing of the report, or the decision to submit the article for publication.

## Results

### Characteristics of the study population

A total of 185,077 participants were included in cooking-related analyses, of whom 23,752 (12.8%) were men, and the baseline mean (SD) age was 51.8 (10.3) (Table 1). For heating-related analyses, 95,228 participants were included, with 29,785 (31.3%) being men, and the baseline mean (SD) age was 51.1 (10.7). At baseline, 60,582 participants (32.7%) and 35,229 participants (37.0%) used solid fuels for cooking and heating in the respective analysis population. Compared with clean fuel users, solid fuel users had a higher proportion of women for cooking but a lower proportion for heating. Whether for cooking or heating, solid fuel users were less likely to use ventilated cookstoves than clean fuel users. There were 230,995 participants included in smoking-related analyses. The baseline mean (SD) age was 51.3 (10.5) years, 106,160 (46.0%) were men, and 72,306 (31.3%) were current smokers or former smokers who quit smoking for illness. The proportions of men and heavy drinkers were higher in smokers than non-smokers.

**Table 1:** Baseline characteristics of the study participants according to cooking and heating fuel types and smoking status.

	Cooking (n = 185,077)^a^		Heating (n = 95,228)^a^		Smoking (n = 230,995)^b^		
	Clean fuels	Solid fuels	Clean fuels	Solid fuels	Never	Former	Current
No. Of participants, n (%)^c^	124,495 (67.3)	60,582 (32.7)	59,999 (63.0)	35,229 (37.0)	150,274 (65.1)	8415 (3.6)	72,306 (31.3)
Age, year (SD)	51.3 (10.3)	52.8 (10.4)	50.5 (10.6)	52.1 (10.9)	50.9 (10.4)	56.6 (10.9)	51.7 (10.3)
Men, n (%)	18,441 (14.8)	5311 (8.8)	13,467 (22.4)	16,318 (46.3)	28,820 (19.2)	7890 (93.8)	69,450 (96.1)
Urban area, n (%)	103,949 (83.5)	11,555 (19.1)	55,228 (92.0)	17,513 (49.7)	117,765 (78.4)	5993 (71.2)	44,885 (62.1)
Middle school and above, n (%)	79,668 (56.6)	14,625 (39.2)	49,880 (80.7)	20,487 (64.3)	95,600 (65.3)	5339 (61.7)	43,738 (57.1)
Married, n (%)	110,911 (89.4)	54,265 (88.9)	53,601 (90.3)	32,065 (89.2)	135,033 (91.8)	7847 (91.4)	67,880 (88.8)
Employed, n (%)	65,313 (57.0)	51,719 (79.5)	33,129 (59.2)	21,640 (53.8)	86,024 (64.2)	5021 (61.0)	56,900 (64.1)
Household income ≥20,000 Yuan/y, n (%)	75,370 (56.7)	19,495 (35.1)	37,348 (59.5)	12,479 (38.5)	91,000 (61.6)	5196 (60.4)	42,048 (55.5)
All or some cookstoves with ventilation, n (%)	111,737 (86.1)	44,424 (82.0)	57,418 (91.6)	25,430 (81.7)	133,055 (88.4)	7530 (87.9)	61,970 (87.1)
Passive smoking, n (%)	95,725 (77.1)	48,893 (80.4)	47,300 (74.6)	24,628 (76.7)	112,344 (68.1)	5359 (75.3)	48,830 (77.4)
Alcohol consumption with pure alcohol intake ≥30g/d, n (%)^d^	3567 (3.1)	2557 (3.6)	2482 (4.4)	1785 (4.6)	4772 (7.3)	2336 (14.4)	22,710 (15.6)
Physical activity, MET-h/d (SD)	20.2 (12.7)	23.4 (13.3)	18.4 (11.0)	18.4 (13.9)	21.0 (13.2)	20.9 (14.7)	21.3 (15.0)
Food consumption daily, n (%)							
Fresh vegetables	121,088 (97.3)	56,828 (93.6)	59,151 (97.7)	33,627 (97.2)	145,741 (97.1)	8177 (97.1)	69,741 (96.2)
Fresh fruits	45,993 (31.3)	3713 (12.3)	33,239 (48.6)	9992 (36.6)	52,161 (31.2)	2410 (31.9)	12,805 (23.4)
Red meat	55,211 (37.5)	8839 (26.1)	31,508 (47.4)	11,440 (39.8)	64,929 (42.6)	3730 (44.2)	31,842 (45.3)
Body mass index, kg/m^2^ (SD)	24.1 (3.3)	23.3 (3.3)	24.3 (3.3)	24.6 (3.6)	24.0 (3.3)	24.4 (3.2)	23.6 (3.2)

MET-h/d indicates metabolic equivalent task hours per day.

All variables were presented as mean (standard deviation) or number (percentage). Baseline characteristics were adjusted for age, sex, and study area, except in the cases where age, sex, or study area was the independent variable of interest.

a Clean fuels refer to electricity, gas, or central heating (for heating only); solid fuels refer to coal and wood.

b Participants who had stopped smoking due to illness were classified as current smokers.

c The numbers in parentheses indicate the percentage of participants who used different fuel types or were in different smoking statuses.

d Former drinkers are those who used to drink at least once weekly but drank less than weekly at baseline and were included in the current category.

### Association of solid fuel use and smoking with all-cause mortality

The median follow-up of years (million person-years) was 12.1 (2.21) in cooking-related analysis, 12.1 (1.12) in heating-related analysis, and 12.0 (2.73) in smoking-related analysis. The deaths recorded during follow-up in the above analysis were 12,725, 7,531, and 18,878, respectively. Based on the Kaplan–Meier curves of all-cause death, the survival probabilities were lower for solid fuel users for cooking or heating, participants using solid fuels but without cookstove ventilation, and current smokers than their counterparts (Figs. S1 and S2).

After adjustment for potential confounders, solid fuel users for cooking had an elevated risk of all-cause death compared with clean fuel users, with HRs (95% CIs) of 1.23 (1.08, 1.41) for men and 1.11 (1.04, 1.18) for women (Table 2). Similar associations were observed for the use of coal and wood. In a joint analysis of fuel type and cookstove ventilation, compared with clean fuel users who reported cooking with ventilation, the HRs (95% CIs) for men who used clean fuels without ventilation, solid fuels with ventilation, and solid fuels without ventilation were 1.12 (0.94, 1.33), 1.24 (1.08, 1.43), and 1.39 (1.16, 1.68), respectively. The association estimates were weaker in women and had no apparent differences between comparison groups.

**Table 2:** Multivariable-adjusted hazard ratios (95% CIs) for all-cause mortality by baseline cooking and heating characteristics and smoking status in men and women separately.

	Men			Women		
	Deaths	Deaths/PYs (/1000)	HRs (95% CIs)	Deaths	Deaths/PYs (/1000)	HRs (95% CIs)
**Cooking (n=185,077)**^a^						
**According to fuel types**						
Clean fuels	1708	7.9	1.00 (Referent)	5478	4.3	1.00 (Referent)
Solid fuels	893	14.7	1.23 (1.08, 1.41)	4646	7.0	1.11 (1.04, 1.18)
Types of solid fuels						
Coal	261	12.6	1.26 (1.03, 1.53)	1315	5.6	1.05 (0.96, 1.15)
Wood	632	15.8	1.23 (1.07, 1.41)	3331	7.8	1.13 (1.06, 1.20)
**According to use of cookstove ventilation**						
Clean fuel use with ventilation	1529	7.8	1.00 (Referent)	4746	4.2	1.00 (Referent)
Clean fuel use without ventilation	179	9.6	1.12 (0.94, 1.33)	732	5.5	1.18 (1.09, 1.29)
Solid fuel use with ventilation	605	14.0	1.24 (1.08, 1.43)	3309	6.7	1.19 (1.12, 1.27)
Solid fuel use without ventilation	288	16.7	1.39 (1.16, 1.68)	1337	7.8	1.19 (1.10, 1.30)
**Heating (n=95,228)**^a^						
Clean fuels	1323	8.4	1.00 (Referent)	2443	4.4	1.00 (Referent)
Solid fuels	2232	11.8	1.29 (1.12, 1.48)	1533	6.9	1.19 (1.07, 1.32)
Types of solid fuels						
Coal	1399	10.8	1.25 (1.08, 1.45)	1065	6.0	1.15 (1.02, 1.30)
Wood	833	14.0	1.35 (1.16, 1.58)	468	10.5	1.24 (1.08, 1.43)
**Smoking (n=230,995)**^b^						
Never	2726	8.1	1.00 (Referent)	6242	4.3	1.00 (Referent)
Former	1057	11.7	1.07 (1.00, 1.15)	116	20.1	1.27 (1.05, 1.53)
Current	8210	10.2	1.41 (1.34, 1.47)	527	16.4	1.40 (1.28, 1.55)

PYs indicate person-years; HR, hazard ratio; CI, confidence interval.

Multivariable models were adjusted for age at baseline, study areas (10 groups for cooking- and smoking-related analyses; 8 groups for heating-related analyses), education (primary school or below, middle or high school, college or university), marital status (married, other status), occupation (agricultural worker, factory worker, other occupations, no occupation), household income (<10,000, 10,000–19,999, and ≥20,000 yuan/year), cookstove with ventilation (yes for all or some stoves, no; adjusted for all analyses except joint analysis of cooking fuel types and use of cookstove ventilation), passive smoking (never lived with smoker, lived with smoker for <20 y, lived with smoker for ≥20 y and exposure <20h/week, lived with smoker for ≥20 y and exposure ≥20h/week), alcohol consumption (never regular or current weekly but not daily, ex-regular, daily <15g/day, 15–29g/day, 30–59g/day, ≥60g/day), physical activity (metabolic equivalent of tasks hours/day), dietary factors (intake frequency of fresh fruits, fresh vegetables, and red meat; the midpoint value of each frequency category was used in the model and treated as continuous), body mass index (BMI, kg/m^2^), menopausal status (pre-menopausal or post-menopausal, only in women), and mutually adjusted for cooking and heating fuel types and smoking status.

a Clean fuels refer to electricity, gas, or central heating (for heating only); solid fuels refer to coal and wood.

b Participants who had stopped smoking due to illness were classified as current smokers.

Solid fuel use for heating was associated with a higher risk of all-cause death, with HRs (95% CIs) of 1.29 (1.12, 1.48) for men and 1.19 (1.07, 1.32) for women (Table 2). Among both men and women, the association estimates were slightly stronger for the use of wood than coal. Similar results were observed in sensitivity analyses (Table S1). For smoking, current and former smokers had a higher mortality risk than never-smokers. The HRs (95% CIs) for current smokers were 1.41 (1.34, 1.47) for men and 1.40 (1.28, 1.55) for women (Table 2).

In subgroup analysis by residence, the association of solid fuel use for cooking or heating with all-cause mortality was similar between urban and rural areas (Table 3). However, the association between smoking and mortality was stronger in urban than rural areas. When cooking and heating fuel use were analyzed jointly, compared with participants both using clean fuels, the HRs (95% CIs) of mortality for those both using solid fuels was 1.35 (1.18, 1.54) in women, higher than that for those who used only solid fuels for cooking or heating (Table S2). The corresponding result was not statistically significant in men. In joint analyses between solid fuel use and tobacco smoking, compared with participants who had never smoked and used clean fuels for cooking, the HRs (95% CIs) of all-cause mortality for ever-smoking/clean fuel users, never-smoking/solid fuel users, and ever-smoking/solid fuel users were 1.43 (1.34, 1.53), 1.28 (1.14, 1.43), and 1.56 (1.42, 1.70) in men, respectively. Similar results were observed in women and the analysis for heating.

**Table 3:** Multivariable-adjusted hazard ratios (95% CIs) for all-cause mortality and life expectancy (LE) difference (95% CI) at age 30 by baseline cooking and heating fuel types and smoking status in urban and rural areas separately.

	Men			Women		
	Deaths	Deaths/PYs (/1000)	HRs (95% CIs)	Deaths	Deaths/PYs (/1000)	HRs (95% CIs)
**Urban area**						
Cooking (n = 115,504)^a^						
Clean fuels	1527	8.1	1.00 (Referent)	4637	4.4	1.00 (Referent)
Solid fuels	209	14.1	1.21 (1.00, 1.46)	993	8.2	1.10 (1.00, 1.20)
Heating (n = 72,741)^a^						
Clean fuels	1229	8.7	1.00 (Referent)	2307	4.5	1.00 (Referent)
Solid fuels	387	9.4	1.31 (1.11, 1.54)	962	5.9	1.17 (1.05, 1.31)
Smoking (n = 168,643)^b^						
Never	1996	8.0	1.00 (Referent)	5134	4.5	1.00 (Referent)
Former	773	12.2	1.08 (0.99, 1.18)	97	20.0	1.27 (1.04, 1.56)
Current	4831	9.8	1.51 (1.43, 1.60)	395	16.8	1.48 (1.33, 1.65)
**Rural area**						
Cooking (n = 69,573)^a^						
Clean fuels	181	6.9	1.00 (Referent)	841	3.7	1.00 (Referent)
Solid fuels	684	15.0	1.29 (1.07, 1.55)	3653	6.7	1.12 (1.03, 1.22)
Heating (n = 22,487)^a^						
Clean fuels	94	5.9	1.00 (Referent)	136	3.3	1.00 (Referent)
Solid fuels	1845	12.5	1.38 (1.04, 1.84)	571	9.9	1.22 (0.91, 1.64)
Smoking (n = 62,352)^b^						
Never	730	8.3	1.00 (Referent)	1108	3.6	1.00 (Referent)
Former	284	10.4	1.06 (0.92, 1.22)	19	20.7	1.40 (0.87, 2.24)
Current	3379	10.8	1.19 (1.10, 1.29)	132	15.3	1.19 (0.98, 1.46)

HR indicates hazard ratio; CI, confidence interval.

Multivariable model was adjusted for the same covariates as in Table 2.

a Clean fuels refer to electricity, gas, or central heating (for heating only); solid fuels refer to coal and wood.

b Participants who had stopped smoking due to illness were classified as current smokers.

### Association of solid fuel use and smoking with LE at age 30

**Fig. 2:**
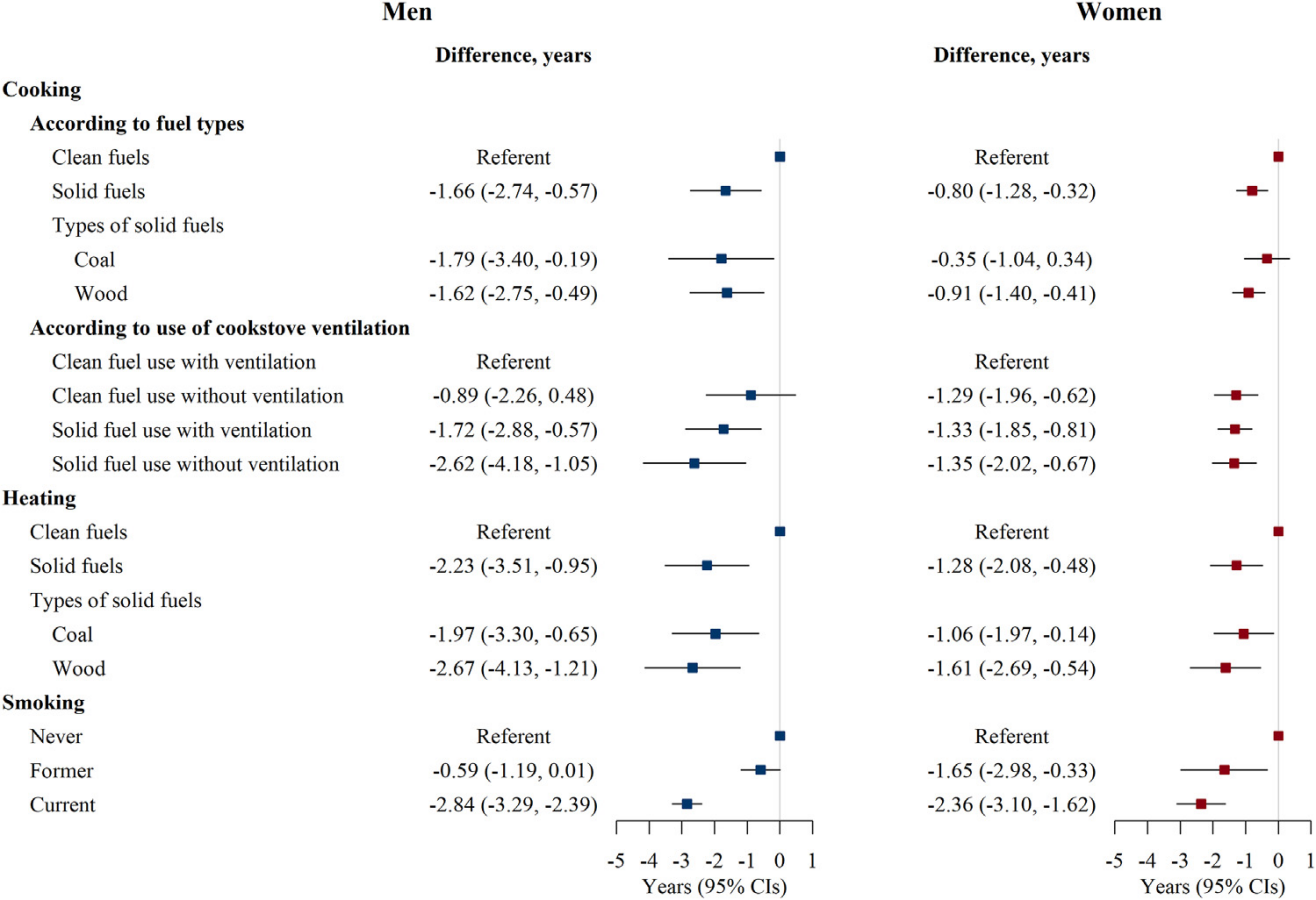
**Life expectancy (LE) difference at age 30 by baseline cooking and heating characteristics and smoking status in men and women separately**. CI indicates confidence interval. Clean fuels refer to electricity, gas, or central heating (for heating only); solid fuels refer to coal and wood. Participants who had stopped smoking due to illness were classified as current smokers.

The difference in LE (95% CI) at age 30 between individuals cooking with solid fuels and clean fuel users was −1.66 (−2.74, −0.57) years for men and −0.80 (−1.28, −0.32) years for women (Fig. 2). Results were comparable when solid fuels were evaluated separately as coal and wood. In the joint analysis of fuel type and ventilation, compared with clean fuel users with cookstove ventilation, the differences in LE (95% CI) for men who used clean fuels without ventilation, solid fuels with ventilation, and solid fuels without ventilation were −0.89 (−2.26, 0.48), −1.72 (−2.88, −0.57), and −2.62 (−4.18, −1.05) years, respectively. The corresponding values for women were −1.29 (−1.96, −0.62), −1.33 (−1.85, −0.81), and −1.35 (−2.02, −0.67) years.

Compared with clean fuel use for heating, the difference in LE (95% CI) for solid fuel use was −2.23 (−3.51, −0.95) in men and −1.28 (−2.08, −0.48) years in women (Fig. 2). Using wood for heating yielded larger life years lost than using coal in both men and women. Sensitivity analyses did not largely change the estimates (Table S1). The difference in LE (95% CI) for current smokers was −2.84 (−3.29, −2.39) years in men compared with never smokers; the corresponding value for women was −2.36 (−3.10, −1.62) years (Fig. 2). All above association results were consistently seen at every age after age 30 (Figs. S3 and S4).

**Fig. 3:**
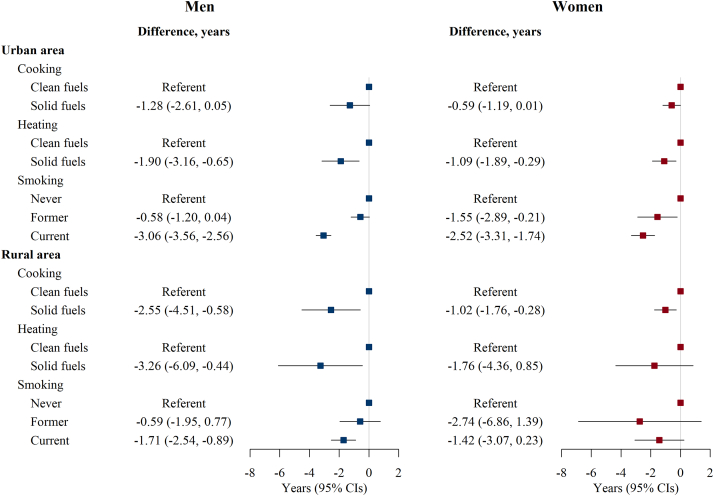
**Life expectancy (LE) difference at age 30 by baseline cooking and heating characteristics and smoking status in urban and rural areas separately**. CI indicates confidence interval. Clean fuels refer to electricity, gas, or central heating (for heating only); solid fuels refer to coal and wood. Participants who had stopped smoking due to illness were classified as current smokers.

In subgroup analysis of urban men, compared with clean fuel users, the difference in LE (95% CI) for solid fuel users was −1.28 (−2.61, 0.05) years for cooking and −1.90 (−3.16, −0.65) years for heating (Fig. 3). Rural men showed a larger difference in LE (95% CI), with the corresponding values of −2.55 (−4.51, −0.58) and −3.26 (−6.09, −0.44) years. Conversely, the difference in LE (95% CI) between never and current smokers in men was larger in urban areas (−3.06; −3.56, −2.56) than in rural areas (−1.71; −2.54, −0.89). The corresponding difference in women was smaller, but the pattern of urban-rural differences for most analyses was similar to that in men.

In the joint analysis of cooking and heating fuel types, compared with participants both using clean fuels, participants both using solid fuels had a greater life-year loss than those using only solid fuels for cooking or heating in women, with the value of −2.45 (−3.55, −1.35) years (Table S2). The corresponding result was not statistically significant in men. When fuel types and smoking were analyzed jointly, compared with participants who had never smoked and used clean fuels, ever-smoking/solid fuel users had the largest negative differences in LE (95% CI) in men, with values of −3.95 (−4.83, −3.07) years for cooking and −4.10 (−5.04, −3.16) years for heating. Ever-smoking/clean fuel users had greater life years lost than never-smoking/solid fuel users. Similar results were observed in women.

## Discussion

In this large prospective cohort study of Chinese adults, participants who used solid fuels for cooking or heating had a lower LE than clean fuel users. Cooking with ventilation could mitigate, to some extent, the adverse impact of solid fuel use on LE in men. In rural men, the use of solid fuels for cooking or heating had a greater impact on LE than smoking, with corresponding reductions in LE at age 30 of 2.55, 3.26, and 1.71 years than their counterparts. In contrast, in urban men, smoking had a greater impact on LE than using solid fuels for cooking or heating, with reductions of 3.06, 1.28, and 1.90 years, respectively. Similar results were observed in women but with a smaller magnitude.

As far as we know, this is the first prospective study quantifying the impact of solid fuel use for cooking and heating on LE. A previous study in Sub-Saharan Africa analyzed the impact of PM_2.5_ from household fuel combustion on LE based on an ecological study design.^6^ The study concluded that a 10% increase in PM_2.5_ concentration was associated with a 0.2 years reduction in LE. However, the study was performed at the country level, and the confounding from other risk factors like smoking could not be controlled, possibly leading to ecological fallacy. In the present study, using solid fuels for heating was associated with a greater life-year loss than cooking. The previous studies which investigated the association of solid fuel use with chronic obstructive pulmonary disease onset, death from cardiovascular diseases, or all-cause mortality also found that solid fuel use for heating had a bigger negative impact than cooking.^5^^,^^18^ A study conducted in northern rural areas of China showed that daily pollutant emissions from heating were higher than those from cooking.^19^ Typically, only a limited number of meals are cooked daily, and the pollutants are short-lived, even cooking with solid fuels. However, during the cold season, when heating always persists throughout the day, and continuous ventilation is not possible, people are constantly exposed to the contaminated environment, resulting in higher inhaled doses of pollutants.

Our results showed that life-year loss related to solid fuel use and smoking was greater in men than in women. Findings from a previous study have shown that the adverse effect related to ambient air pollution was also more pronounced in men than women.^20^ From a physiological perspective, women's reduced airways diameter and lung volume could result in lower peak expiratory flow and vital capacity, which may lead to fewer air pollutants inhaled by women.^21^ However, directly measured exposure level to air pollutants was not currently available in our study. Future studies with this information are required to confirm this hypothesis. For smoking, results from neuroimaging studies indicated that smoking activates men's reward pathways more than women's, which confirmed the established notion that men smoke for the reinforcing drug effect of cigarettes. Thus, men are more susceptible to addiction to smoking and vulnerable to its toxic effects.^22^

In the joint analysis of cooking fuels and the use of ventilation, we observed that among men who used solid fuels, the years of life lost were greater among those who did not use ventilation than those who used ventilation, suggesting that ventilation could partly, but not entirely, offset the harmful effects of solid fuel use. Several previous studies have also shown that ventilation could mitigate the increased risk of all-cause mortality and morbidity and mortality risk from cardiopulmonary diseases.^4^^,^^23^, ^24^, ^25^ However, such a difference in the years of life lost due to use of ventilation was not seen among women who used solid fuels in this study. As we discussed earlier, in the context of the relatively small harmful effect of solid fuels on women, the protective role of ventilation was relatively insignificant, whereas further research is needed. Additionally, we found that, even among clean fuel users, not using ventilation was associated with reduced LE in women. The small number of deaths and insufficient statistical power may result in a small reduction that was not statistically significant among clean fuel users in men. Our finding was similar to the previous study, suggesting that clean fuel is not entirely exempt from producing pollutants. Ventilation can also protect people who persistently use clean fuels by lowering the risk of mortality.^4^^,^^26^

In the subgroup analysis by residence, we observed that the LE reduction related to solid fuel use was greater in rural population, whereas that related to smoking was greater in urban population. One possible explanation is that rural residents may have more frequent or intense exposures during cooking or heating than urban residents.^5^ Unfortunately, we did not collect detailed information on the frequency and cumulative duration of exposure to cooking and heating. Also, possibly restricted by the affordability of energy resources, as shown in the CKB population,^27^ rural residents preferred to use biomass like wood and crop residue as fuel, which contains higher volatile matter content and lower ash content than coal.^28^ Combustion of biomass emits more particulate matter than coal and poses a greater health risk.^29^^,^^30^ In the present study, participants using wood for cooking or heating had a greater life-year loss than those using coal, which partly explains the greater harmful effects of solid fuel use in rural areas. Regarding smoking, a previous study conducted in the CKB population showed that earlier initiation of smoking was associated with an increased risk of death from cardiopulmonary diseases and cancer, as well as all-cause mortality.^10^ Tobacco consumption became widespread earlier in urban than in rural areas. Hence, the “full effect” of smoking has been manifested in urban areas.

The main strengths of this study include the large sample size, prospective design with a long follow-up period, and low rate of loss to follow-up. It enabled us to minimize the confounding by excluding participants exposed to other air pollution sources and smokers when analyzing the effect of solid fuel use for cooking or heating. The large sample size also allowed us to estimate LE by sex and urban and rural areas. Furthermore, in addition to the relative risk, we also reported an absolute measure, specifically, how many life years participants would lose due to using solid fuels. We took the adverse impact of smoking as a reference to help better understand the harmful effects of solid fuels.

Several limitations should be noted when interpreting the results of this study. First, solid fuel use and smoking were self-reported. The possible misclassification may bias the association toward the null. However, we compared responses to the same questions between the baseline and a resurvey shortly after the baseline in a random sample of the CKB population. The reproducibility of fuel type and smoking was reasonably good.^5^ Second, we only recorded the fuel use and smoking status once at baseline; possible changes in these exposures could not be considered. However, the hazardous impact of solid fuel use and smoking are chronic, and health deterioration occurring during follow-up is more likely to result from early long-term exposure. Moreover, the use of baseline exposure status may also help avoid reverse causation resulting from exposure changes after health deterioration. Third, we endeavored to refine our assessment of solid fuel exposure by excluding participants with mixed use of fuel types before baseline. However, those who reported switching from solid to clean fuels were retained due to the non-negligible number of participants. In our sensitivity analysis, we excluded the participants who had stopped using solid fuels for <10 years from the referent group, and the results did not alter notably.^31^

In this large, prospective cohort study of the Chinese population, household use of solid fuels for cooking or heating was associated with lower LE. Using solid fuels for cooking without ventilation and heating with solid fuels had roughly similar impacts on LE as smoking. In rural populations, solid fuel-related harms were even greater than that from smoking. Our findings highlight the complexity and diversity of the factors affecting LE in less-developed countries. Besides the lifestyle risk factors of global health importance, such as smoking, factors unique to less-developed populations, like solid fuel use, should not be ignored. Addressing these risk factors to improve health outcomes and health equity will heavily depend on sectors other than health through boosting the infrastructure of clean energy supply.

## Contributors

JL and LL conceived and designed the study, contributed to the interpretation of the results and critical revision of the manuscript for valuable intellectual content. LL, ZC, and JC: as the members of the CKB steering committee, designed and supervised the conduct of the whole study, obtained funding, and together with CY, YG, DJYS, PP, LY, YC, HD, DSchmidt, RS, and KK, acquired the CKB data. QS and DS accessed, verified, and analyzed the data. QS drafted the manuscript. All authors had access to the data and have read and approved the final manuscript. The corresponding author attests that all listed authors meet authorship criteria and that no others meeting the criteria have been omitted. JL and LL are the guarantors.

## Data sharing statement

Details of how to access China Kadoorie Biobank data and details of the data release schedule are available from www.ckbiobank.org/site/Data+Access.

## Declaration of interests

We declare no competing interests.

## Appendix A Supplementary data

Supplementary appendix

Abstract_chineseversion
