# Supplementary material for: Impacts of solid fuel use versus smoking on life expectancy at age 30 years in the rural and urban Chinese population: a prospective cohort study
Source: Lancet Reg Health West Pac. 2023 Feb 13;32:100705. doi: 10.1016/j.lanwpc.2023.100705 (PMC9942113; doi:10.1016/j.lanwpc.2023.100705)
Supplement: Supplementary appendix [file mmc1.docx]

**Supplementary Material**

[**Members of the China Kadoorie Biobank collaborative group** 2](#_Toc122524711)

[**Statistical method used for estimating years of life lost (YLL)** 3](#_Toc122524712)

[**References** 5](#_Toc122524713)

[**Table S1. Sensitivity analyses of adjusted hazard ratios (95% CIs) for all-cause mortality and life expectancy (LE) difference (95% CI) at age 30 by baseline cooking and heating fuel types** 6](#_Toc122524714)

[**Table S2. Multivariable-adjusted hazard ratios (95% CIs) for all-cause mortality and life expectancy (LE) difference (95% CI) at age 30 by joint categories of household fuel types and smoking status** 7](#_Toc122524715)

[**Figure S1. Kaplan-Meier probabilities of all-cause death during follow-up by baseline cooking fuel types and combined use of cookstove ventilation in men and women separately** 9](#_Toc122524716)

[**Figure S2. Kaplan-Meier probabilities of all-cause death during follow-up by baseline heating fuel types and smoking status in men and women separately** 10](#_Toc122524717)

[**Figure S3. Years of life lost after age 30 by baseline cooking fuel types and combined use of cookstove ventilation in men and women separately** 11](#_Toc122524718)

[**Figure S4. Years of life lost after age 30 by baseline heating fuel types and smoking status in men and women separately** 12](#_Toc122524719)

**Members of the China Kadoorie Biobank collaborative group**

**International Steering Committee:** Junshi Chen, Zhengming Chen (PI), Robert Clarke, Rory Collins, Yu Guo, Liming Li (PI), Jun Lv, Richard Peto, Robin Walters. **International Co-ordinating Centre, Oxford:** Daniel Avery, Derrick Bennett, Ruth Boxall, Sue Burgess, Ka Hung Chan, Yumei Chang, Yiping Chen, Zhengming Chen, Johnathan Clarke; Robert Clarke, Huaidong Du, Ahmed Edris Mohamed, Zammy Fairhurst-Hunter, Hannah Fry, Simon Gilbert, Alex Hacker, Mike Hill, Michael Holmes, Pek Kei Im, Andri Iona, Maria Kakkoura, Christiana Kartsonaki, Rene Kerosi, Kuang Lin, Mohsen Mazidi, Iona Millwood, Sam Morris, Qunhua Nie, Alfred Pozarickij, Paul Ryder, Saredo Said, Sam Sansome, Dan Schmidt, Paul Sherliker, Rajani Sohoni, Becky Stevens, Iain Turnbull, Robin Walters, Lin Wang, Neil Wright, Ling Yang, Xiaoming Yang, Pang Yao.

**National Co-ordinating Centre, Beijing:** Yu Guo, Xiao Han, Can Hou, Jun Lv, Pei Pei, Chao Liu, Canqing Yu, Qingmei Xia. **10 Regional Co-ordinating Centres: Qingdao CDC:** Zengchang Pang, Ruqin Gao, Shanpeng Li, Haiping Duan, Shaojie Wang, Yongmei Liu, Ranran Du, Yajing Zang, Liang Cheng, Xiaocao Tian, Hua Zhang, Yaoming Zhai, Feng Ning, Xiaohui Sun, Feifei Li. **Licang CDC:** Silu Lv, Junzheng Wang, Wei Hou. **Heilongjiang Provincial CDC:** Wei Sun, Shichun Yan, Xiaoming Cui. **Nangang CDC:** Chi Wang, Zhenyuan Wu,Yanjie Li, Quan Kang. **Hainan Provincial CDC:** Huiming Luo, Tingting Ou. **Meilan CDC:** Xiangyang Zheng, Zhendong Guo, Shukuan Wu, Yilei Li, Huimei Li. **Jiangsu Provincial CDC:** Ming Wu, Yonglin Zhou, Jinyi Zhou, Ran Tao, Jie Yang, Jian Su. **Suzhou CDC:** Fang Liu, Jun Zhang, Yihe Hu, Yan Lu, Liangcai Ma, Aiyu Tang, Shuo Zhang, Jianrong Jin, Jingchao Liu. **Guangxi Provincial CDC:** Mei Lin, Zhenzhen Lu. **Liuzhou CDC:** Lifang Zhou, Changping Xie, Jian Lan,Tingping Zhu,Yun Liu, Liuping Wei, Liyuan Zhou, Ningyu Chen, Yulu Qin, Sisi Wang. **Sichuan Provincial CDC:** Xianping Wu, Ningmei Zhang, Xiaofang Chen, Xiaoyu Chang. **Pengzhou CDC:** Mingqiang Yuan, Xia Wu, Xiaofang Chen, Wei Jiang, Jiaqiu Liu, Qiang Sun. **Gansu Provincial CDC:** Faqing Chen, Xiaolan Ren, Caixia Dong. **Maiji CDC:** Hui Zhang, Enke Mao, Xiaoping Wang, Tao Wang, Xi zhang. **Henan Provincial CDC:** Kai Kang, Shixian Feng, Huizi Tian, Lei Fan. **Huixian CDC:** XiaoLin Li, Huarong Sun, Pan He, Xukui Zhang. **Zhejiang Provincial CDC:** Min Yu, Ruying Hu, Hao Wang. **Tongxiang CDC**: Xiaoyi Zhang, Yuan Cao, Kaixu Xie, Lingli Chen, Dun Shen. **Hunan Provincial CDC:** Xiaojun Li, Donghui Jin, Li Yin, Huilin Liu, Zhongxi Fu. **Liuyang CDC:** Xin Xu, Hao Zhang, Jianwei Chen,Yuan Peng, Libo Zhang, Chan Qu.

**Statistical method used for estimating years of life lost (YLL)**

The calculation of years of life lost involves three steps as follows, and the application of each step is described in detail below:^1,2^

1. First, the survival curve is predicted for each individual based on the Royston-Parmar parametric model and averaged over all individuals;
2. Second, residual life expectancy is estimated as the area under the survival curve by integrating the curve up to age 100, conditional on surviving at ages 30 to 100 (1-year intervals);
3. Third, the years of life lost and 95% confidence intervals are calculated as the difference between the areas under the survival curves of different exposure categories of interest.

The Royston-Parmar parametric model is fitted based on the log cumulative hazard (*H*) scale, with age as the timescale.^3,4^ For individual *i*, the cumulative hazard at age *t* is given by:

$H\left( t | x_{i} \right)=H_{0}(t)\times exp(\beta\times x_{i})$ [1]

Where $H_{0}(t)$ is the baseline cumulative hazard at age *t*; $\beta$ is the vector of coefficients for all-cause mortality; $x_{i}$ is the vector of covariates for individual *i*. Expression [1] is estimated by replacing baseline cumulative hazard and coefficients with their estimates. In Royston-Parmar parametric model, baseline cumulative hazard is a restricted cubic spline function of the natural logarithm of age.

The survival probability at age *t* for individual *i* with covariates $x_{i}$ is the usual simple transformation of the cumulative hazard:

$S(t|x_{i})=\exp\left( -H\left( t | x_{i} \right) \right)$ [2]

Taking cooking fuel use-related analysis as an example, at the population level, marginal survival probabilities of individuals using clean fuels or solid fuels are estimated by assuming all individuals in the analysis data using clean fuels or solid fuels respectively and then averaged.

When calculating the life expectancy at age *T*, conditional survival probability at age *t* given survival to age *T* should be used, which is estimated as the marginal survival probability at age *t* divided by that at age *T*:

$S(t|T)=\exp\left( -H(t) \right)/\exp\left( -H(T) \right)$ [3]


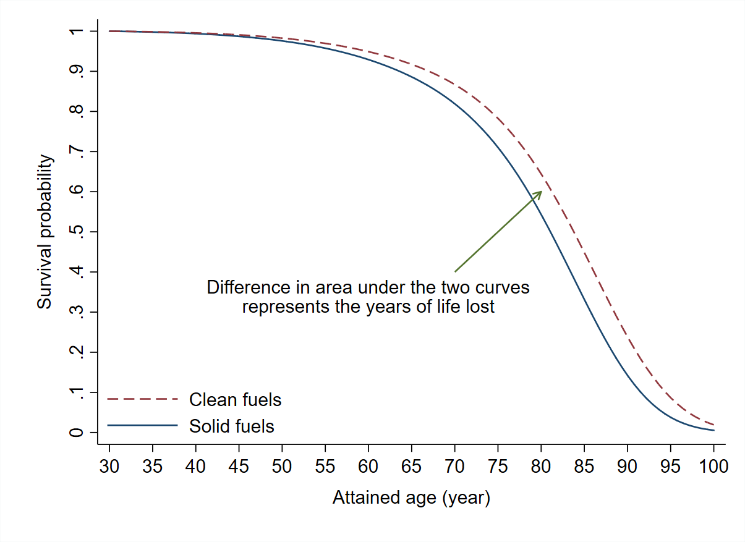


Supposing the two curves in the above figure represent the survival function of a cohort population at 30-year old who used clean fuels (red line) or solid fuels (blue line) at baseline, the years of life lost of the population using solid fuels at age 30 are estimated from the difference in the area under the two curves, i.e.:

$YLL=\int_{30}^{100} (S_{c}\left( u \right)-S_{s}\left( u \right))du$ [4]

Where $S_{c}\left( u \right)$ and $S_{s}\left( u \right)$ represent conditional survival probabilities at age u of the cohort population using clean fuels and solid fuels, respectively.

**References**

1. Chudasama YV, Khunti K, Gillies CL, et al. Healthy lifestyle and life expectancy in people with multimorbidity in the UK Biobank: A longitudinal cohort study. *PLoS medicine* 2020; **17**(9): e1003332.

2. Chudasama YV, Khunti KK, Zaccardi F, et al. Physical activity, multimorbidity, and life expectancy: a UK Biobank longitudinal study. *BMC medicine* 2019; **17**(1): 108.

3. Nelson CP, Lambert PC, Squire IB, Jones DR. Flexible parametric models for relative survival, with application in coronary heart disease. *Stat Med* 2007; **26**(30): 5486-98.

4. Lambert PC, Royston P. Further development of flexible parametric models for survival analysis. *Stata Journal* 2009; **9**(2): 265-90.

**Table S1. Sensitivity analyses of adjusted hazard ratios (95% CIs) for all-cause mortality and life expectancy (LE) difference (95% CI) at age 30 by baseline cooking and heating fuel types**

|  | **Men** | | |  | **Women** | | |
| --- | --- | --- | --- | --- | --- | --- | --- |
|  | Deaths | HRs (95% CIs)^‡^ | Difference, years (95% CIs) |  | Deaths | HRs (95% CIs)^‡^ | Difference, years (95% CIs) |
| **Excluding participants who had stopped using solid fuels for <10 years from the referent group** | | | | | | | |
| Cooking^†^ |  |  |  |  |  |  |  |
| Clean fuels | 1,517 | 1.00 (Referent) | Referent |  | 4,403 | 1.00 (Referent) | Referent |
| Solid fuels | 893 | 1.23 (1.07, 1.40) | -1.58 (-2.70, -0.47) |  | 4,646 | 1.13 (1.06, 1.21) | -0.93 (-1.45, -0.41) |
| Heating^†^ |  |  |  |  |  |  |  |
| Clean fuels | 1,096 | 1.00 (Referent) | Referent |  | 2,012 | 1.00 (Referent) | Referent |
| Solid fuels | 2,232 | 1.32 (1.12, 1.54) | -2.48 (-3.95, -1.01) |  | 1,533 | 1.14 (1.02, 1.28) | -0.98 (-1.84, -0.12) |
| **Excluding areas with low solid fuel usage**^*^ | | | | | | | |
| Cooking^†^ |  |  |  |  |  |  |  |
| Clean fuels | 1,128 | 1.00 (Referent) | Referent |  | 3,781 | 1.00 (Referent) | Referent |
| Solid fuels | 893 | 1.22 (1.07, 1.39) | -1.67 (-2.85, -0.49) |  | 4,645 | 1.11 (1.04, 1.18) | -0.79 (-1.29, -0.29) |
| Heating^†^ |  |  |  |  |  |  |  |
| Clean fuels | 1,256 | 1.00 (Referent) | Referent |  | 2,278 | 1.00 (Referent) | Referent |
| Solid fuels | 2,231 | 1.27 (1.11, 1.46) | -2.15 (-3.43, -0.86) |  | 1,533 | 1.19 (1.07, 1.32) | -1.28 (-2.08, -0.48) |

HR indicates hazard ratio; CI, confidence interval.

^*^For cooking-related analyses, two urban areas, including Qingdao and Harbin, were excluded because they had few participants (Qingdao: 0.47%; Harbin: 1.50%) using solid fuels for cooking. For heating-related analyses, an urban area, Suzhou, was excluded because it had few participants (0.05%) using solid fuels for winter heating.

^†^Clean fuels refer to electricity, gas, or central heating (for heating only); solid fuels refer to coal and wood.

^‡^Multivariable models were adjusted for age at baseline, study areas, education (primary school or below, middle or high school, college or university), marital status (married, other status), occupation (agricultural worker, factory worker, other occupations, no occupation), household income (<10,000, 10,000-19,999, and ≥20,000 yuan/year), cookstove with ventilation (yes for all or some stoves, no), smoking status (never/occasional, former), passive smoking (never lived with smoker, lived with smoker for <20y, lived with smoker for ≥20y and exposure <20h/week, lived with smoker for ≥20y and exposure ≥20h/week), alcohol consumption (never regular or current weekly but not daily, ex-regular, daily <15g/day, 15-29g/day, 30-59g/day, ≥60g/day), physical activity (metabolic equivalent of tasks hours/day), dietary factors (intake frequency of fresh fruit, fresh vegetable, and meat; the midpoint value of each frequency category was used in the model and treated as continuous), body mass index (BMI, kg/m^2^), menopausal status (pre-menopausal or post-menopausal, only in women), and mutually adjusted for cooking and heating fuel types.

**Table S2. Multivariable-adjusted hazard ratios (95% CIs) for all-cause mortality and life expectancy (LE) difference (95% CI) at age 30 by joint categories of household fuel types and smoking status**

|  | **Men** | | |  | **Women** | | |
| --- | --- | --- | --- | --- | --- | --- | --- |
|  | Deaths | HRs (95% CIs)^*^ | Difference, years (95% CIs) |  | Deaths | HRs (95% CIs)^*^ | Difference, years (95% CIs) |
| **Cooking fuel types × heating fuel types (n=158,890)^†^** | | | | | | | |
| Both clean fuels^§^ | 691 | 1.00 (Referent) | Referent |  | 2,147 | 1.00 (Referent) | Referent |
| Cook - clean × heat - solid | 231 | 1.28 (1.05, 1.57) | -1.97 (-3.63, -0.30) |  | 961 | 1.15 (1.03, 1.27) | -1.12 (-1.99, -0.24) |
| Cook - solid × heat - clean | 48 | 0.93 (0.64, 1.34) | 0.58 (-2.27, 3.43) |  | 338 | 1.07 (0.92, 1.25) | -0.56 (-1.85, 0.72) |
| Both solid fuels | 883 | 1.30 (0.98, 1.74) | -2.10 (-4.41, 0.22) |  | 6,946 | 1.35 (1.18, 1.54) | -2.45 (-3.55, -1.35) |
| **Cooking fuel types × smoking status (n=234,891)**^‡^ | | | | | | | |
| Clean fuels |  |  |  |  |  |  |  |
| Never smoker^§^ | 1,212 | 1.00 (Referent) | Referent |  | 5,392 | 1.00 (Referent) | Referent |
| Ever smoker | 3,906 | 1.43 (1.34, 1.53) | -3.17 (-3.83, -2.51) |  | 534 | 1.38 (1.26, 1.52) | -2.53 (-3.32, -1.75) |
| Solid fuels |  |  |  |  |  |  |  |
| Never smoker | 647 | 1.28 (1.14, 1.43) | -2.17 (-3.18, -1.16) |  | 4,561 | 1.13 (1.06, 1.20) | -0.93 (-1.40, -0.45) |
| Ever smoker | 2,782 | 1.56 (1.42, 1.70) | -3.95 (-4.83, -3.07) |  | 849 | 1.51 (1.37, 1.66) | -3.24 (-4.07, -2.41) |
| **Heating fuel types × smoking status (n=159,593)**^‡^ | | | | | | | |
| Clean fuels |  |  |  |  |  |  |  |
| Never smoker^§^ | 925 | 1.00 (Referent) | Referent |  | 2,355 | 1.00 (Referent) | Referent |
| Ever smoker | 2,859 | 1.42 (1.32, 1.53) | -3.21 (-3.95, -2.46) |  | 435 | 1.40 (1.26, 1.56) | -2.53 (-3.44, -1.61) |
| Solid fuels |  |  |  |  |  |  |  |
| Never smoker | 1,748 | 1.29 (1.16, 1.43) | -2.32 (-3.29, -1.36) |  | 1,521 | 1.23 (1.11, 1.36) | -1.57 (-2.38, -0.77) |
| Ever smoker | 6,832 | 1.57 (1.42, 1.72) | -4.10 (-5.04, -3.16) |  | 110 | 1.65 (1.35, 2.02) | -3.79 (-5.47, -2.12) |

HR indicates hazard ratio; CI, confidence interval.

Clean fuels refer to electricity, gas, or central heating (for heating only); solid fuels refer to coal and wood.

^*^Multivariable models were adjusted for age at baseline, study areas, education, marital status, occupation, household income, cook-stove ventilation, passive smoking, alcohol consumption, physical activity, dietary factors (intake frequency of fresh fruit, fresh vegetable, and meat), body mass index, menopausal status (only in women), and another exposure that was not included in the joint grouping.

^†^Participants used for both cooking- and heating-related analyses in the primary analysis were included in this joint analysis.

^‡^Compared with the cooking- and heating-related analyses in the primary analysis, participants who smoked at baseline were retained in this joint analysis with smoking, leaving 234,891 participants (67,621 men and 167,270 women) for cooking-related analyses and 159,593 (91,835 men and 67,758 women) for heating-related analyses.

^§^Common reference group.

**
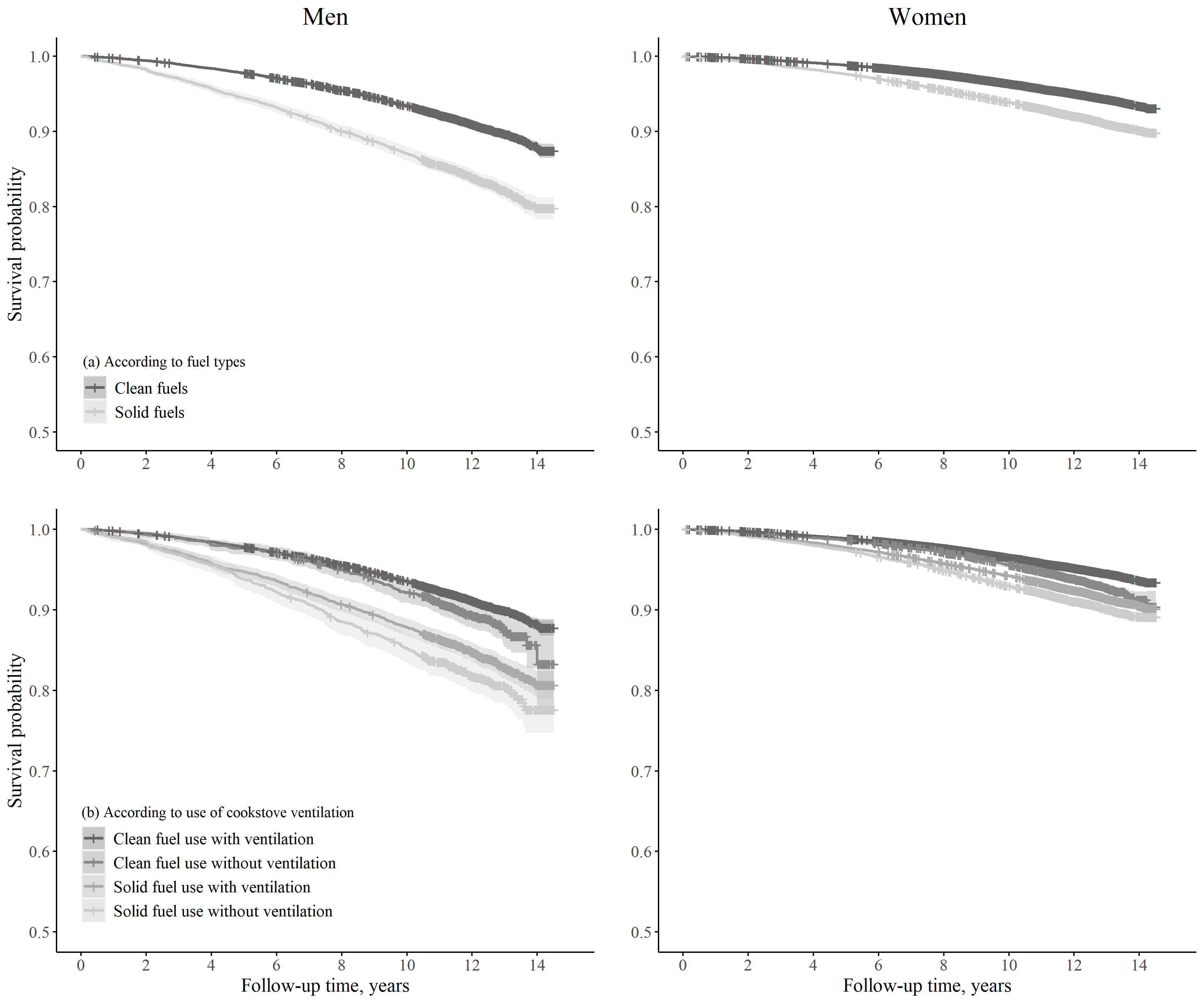
**

**Figure S1. Kaplan-Meier probabilities of all-cause death during follow-up by baseline cooking fuel types and combined use of cookstove ventilation in men and women separately**

Clean fuels refer to electricity and gas; solid fuels refer to coal and wood. Participants used for making the Kaplan-Meier plots were the same as those used in the primary cooking-related analysis.


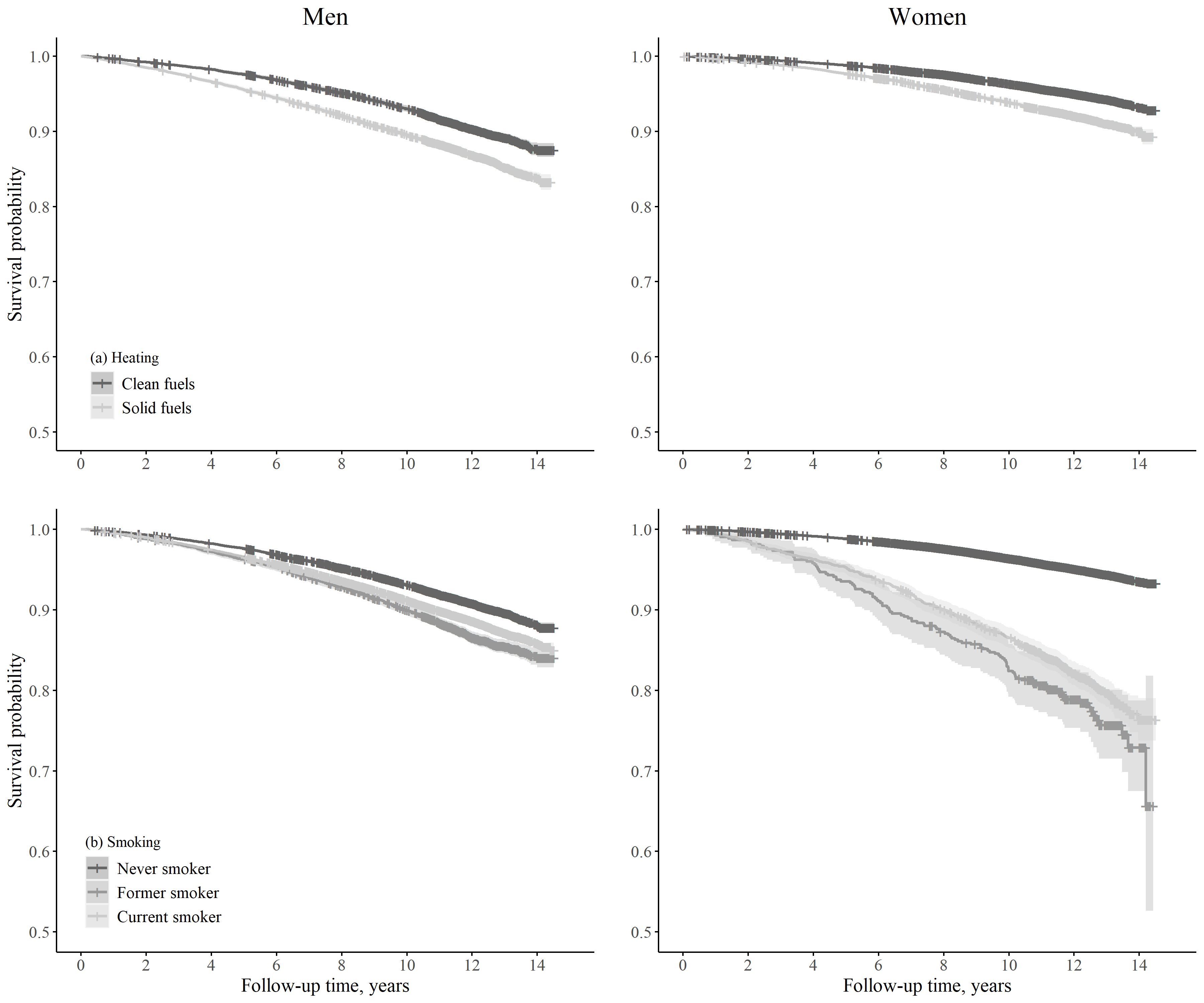


**Figure S2. Kaplan-Meier probabilities of all-cause death during follow-up by baseline heating fuel types and smoking status in men and women separately**

Clean fuels refer to electricity, gas, or central heating; solid fuels refer to coal and wood. Participants who had stopped smoking due to illness were classified as current smokers. Participants used for making the Kaplan-Meier plots by heating fuel types and smoking status were the same as those used in the primary heating-, and smoking-related analysis, respectively.

**
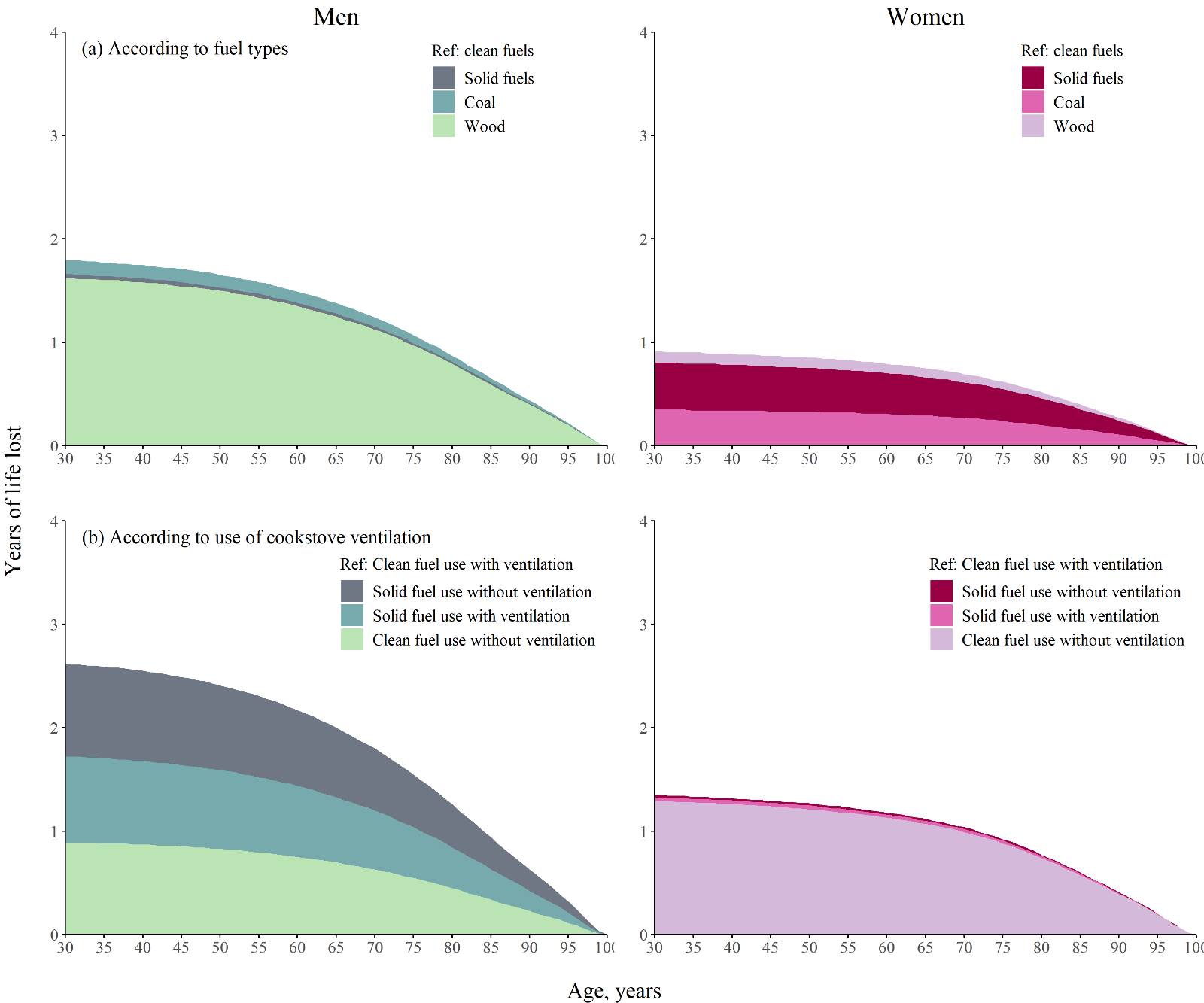
**

**Figure S3. Years of life lost after age 30 by baseline cooking fuel types and combined use of cookstove ventilation in men and women separately**

Clean fuels refer to electricity and gas; solid fuels refer to coal and wood.


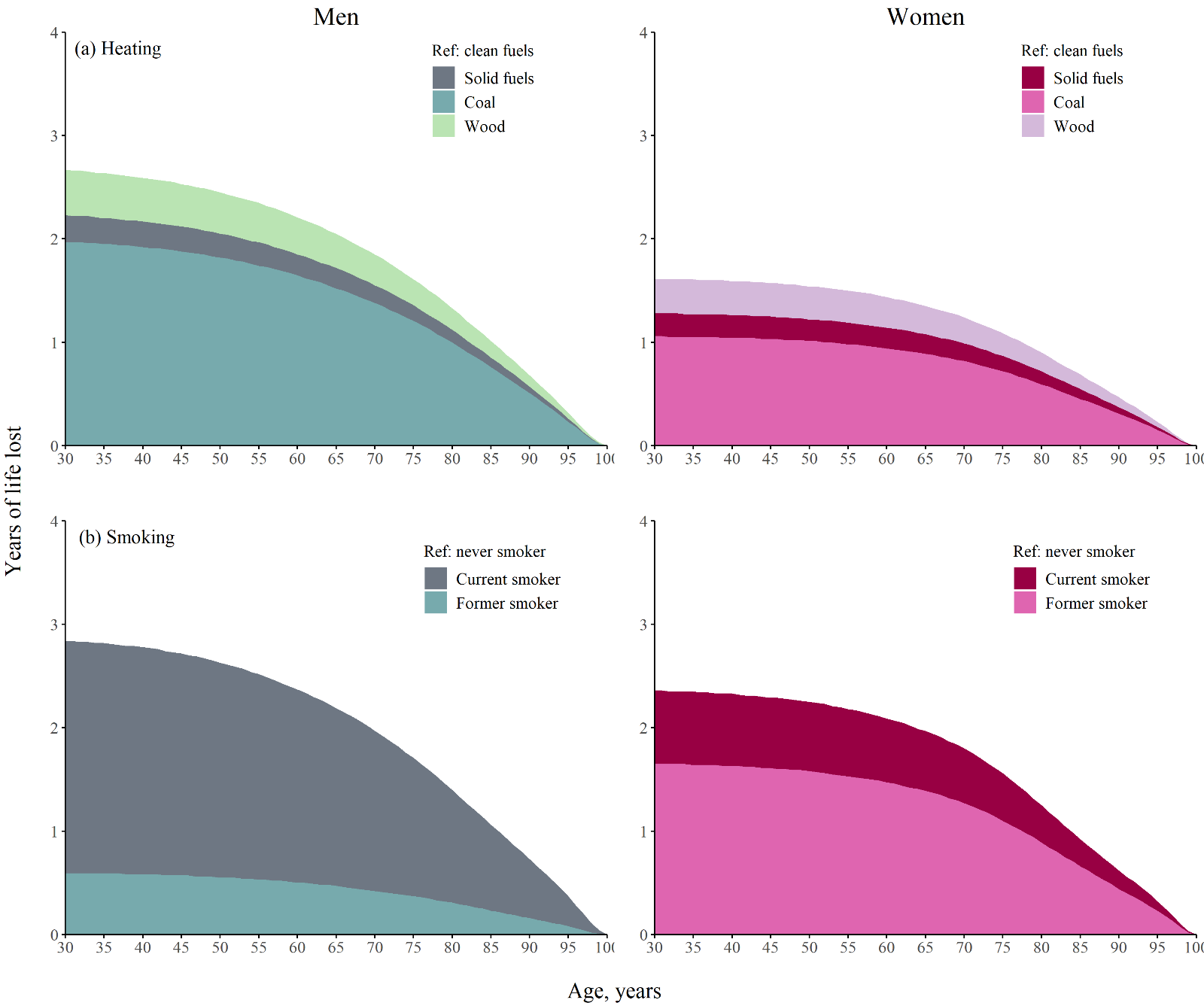


**Figure S4. Years of life lost after age 30 by baseline heating fuel types and smoking status in men and women separately**

Clean fuels refer to electricity, gas, or central heating; solid fuels refer to coal and wood. Participants who had stopped smoking due to illness were classified as current smokers.
