# Supplementary material for: Impacts of solid fuel use versus smoking on life expectancy at age 30 years in the rural and urban Chinese population: a prospective cohort study
Source: Lancet Reg Health West Pac. 2023 Feb 13;32:100705. doi: 10.1016/j.lanwpc.2023.100705 (PMC9942113; doi:10.1016/j.lanwpc.2023.100705)
Supplement: Abstract_chineseversion [file mmc2.docx]

**使用固体燃料与吸烟对中国城乡人口30岁时期望寿命的影响研究：一项前瞻性队列研究**

**摘要**

**背景：**在欠发达的国家或地区，使用固体燃料对期望寿命（life expectancy）的影响尚不清楚。本研究旨在评估室内使用固体燃料对中国城市和农村人群期望寿命的影响大小，并以吸烟的影响作为参照。

**方法：**在该研究中，我们使用了中国慢性病前瞻性研究（China Kadoorie Biobank）的数据。在剔除基线时患有冠心病、中风或癌症的研究对象后，本研究共纳入484,915名30-79岁成年人。我们分别对使用固体燃料做饭、使用固体燃料取暖和吸烟的效应进行了独立分析，并在分析其中一项暴露因素的效应时排除了有其他两项暴露因素者。本研究中，固体燃料指煤炭和木材，清洁燃料指电、煤气和集中供暖。我们使用灵活参数生存分析模型，即Royston-Parmar模型来估计各暴露因素与全因死亡之间的关联，并同时估计了不同暴露人群30岁时的期望寿命及其差异大小。

**结果：**做饭、取暖和吸烟相关的分析共分别纳入了185,077、95,228和230,995名研究对象。在约12.1年的中位随访时间内，各分析人群中分别记录到了12,725、7,531和18,878例死亡事件的发生。与做饭时使用清洁燃料并且通风者相比，男性中使用固体燃料但通风者以及使用固体燃料且不通风者在30岁时的期望寿命差异（95% confidence intervals [CIs]）为-1.72（-2.88，-0.57）岁和-2.62（-4.16，-1.05）岁，女性中对应的差异数值为-1.33（-1.85，-0.81）岁和-1.35（-2.02，-0.67）岁。在取暖相关的分析中，与使用清洁燃料者相比，男性中使用固体燃料者30岁时的期望寿命差异（95% CI）为-2.23（-3.51，-0.95），女性中为-1.28（-2.08，-0.48）岁。在农村人群中，与使用固体燃料做饭或是取暖相关的期望寿命降低比吸烟相关的期望寿命降低更为明显，男性中对应的期望寿命差异数值（95% CIs）分别为-2.55（-4.51，-0.58）、-3.26（-6.09，0.44）和-1.71（-2.54，-0.89）。相反，在城市男性中，与吸烟（-3.06；95% CI: -3.56，-2.56）相关的期望寿命降低幅度明显大于使用固体燃料做饭（-1.28；95% CI: -2.61，0.05）或取暖（-1.90；95% CI: -3.16，-0.65）。女性中的观察结果类似，但期望寿命差异数值的绝对值均低于男性。

**解释：**在该中国人群队列中，室内使用固体燃料对期望寿命的危害在农村居民中要大于吸烟。相反，在城市居民中，吸烟的负面影响要大于使用固体燃料。我们的研究结果强调了欠发达地区人口中期望寿命影响因素的复杂性和多样性。

*This translation in Chinese was submitted by the authors and we reproduce it as supplied. It has not been peer reviewed. Our editorial processes have only been applied to the original abstract in English, which should serve as reference for this manuscript.*
